# Pancreaticopleural Fistula: A Rare Presentation and a Rare Complication

**DOI:** 10.7759/cureus.4984

**Published:** 2019-06-24

**Authors:** Ahmad Ramahi, Kanana Mohammad Aburayyan, Tamer S Said Ahmed, Vyas Rohit, Mohammad Taleb

**Affiliations:** 1 Internal Medicine, University of Toledo Medical Center, Toledo, USA; 2 Pulmonary / Critical Care Medicine, University of Toledo Medical Center, Toledo, USA

**Keywords:** pancreaticopleural fistula, pleural effusion, pancreatitis

## Abstract

Pancreaticopleural fistula (PPF) is a rare complication of pancreatitis that requires a high index of clinical suspicion as patients typically present with pulmonary symptoms related to the pleural effusion rather than pancreatitis. Diagnosis is made by detection of amylase in the pleural fluid. Magnetic resonance cholangiopancreatography can aid in visualizing the fistula. We present a case of massive left pleural effusion secondary to a PPF due to acute on chronic pancreatitis.

## Introduction

Pancreaticopleural fistula (PPF) is a rare diagnosis that occurs approximately in 0.4% of patients with pancreatitis [1]. Due to chronic pancreatic inflammation, an abnormal connection known as fistula is formed between the pancreatic duct and the pleural space, leading to pancreatic secretion drainage into the pleura causing pleural effusion that is high in amylase. Patients typically present with pulmonary symptoms related to the pleural effusion rather than to pancreatitis leading to delay in diagnosis [1]. Once the diagnosis is confirmed, treatment is usually directed to obliterate the fistula between the pancreas and the pleural. 

## Case presentation

A 65-year old male patient with a past medical history of chronic obstructive pulmonary disease (COPD), chronic alcoholic pancreatitis, and squamous cell carcinoma of the oropharynx status post-chemoradiotherapy in 2014 presented to the hospital due to abdominal pain and shortness of breath. His abdominal pain was in the epigastric area, sharp in nature, radiating to the back, associated with nausea, and constipation. The patient had no fever, chills, chest pain, cough, or vomiting. 

On physical exam, blood pressure 117/60 mmHg, heart rate of 86 beat per minute, respiratory rate of 16 breaths per minute, temperature 36.6°C, oxygen saturation 95% on room air, the patient was alert and oriented. Heart examination showed a regular rate and rhythm with normal S1 and S2. Lung examination showed decreased breath sounds with dullness on percussion of the left side of the chest. Abdominal examination was significant for epigastric tenderness and hypoactive bowel sounds. Other systems’ examination was unremarkable. 

**Figure 1:**
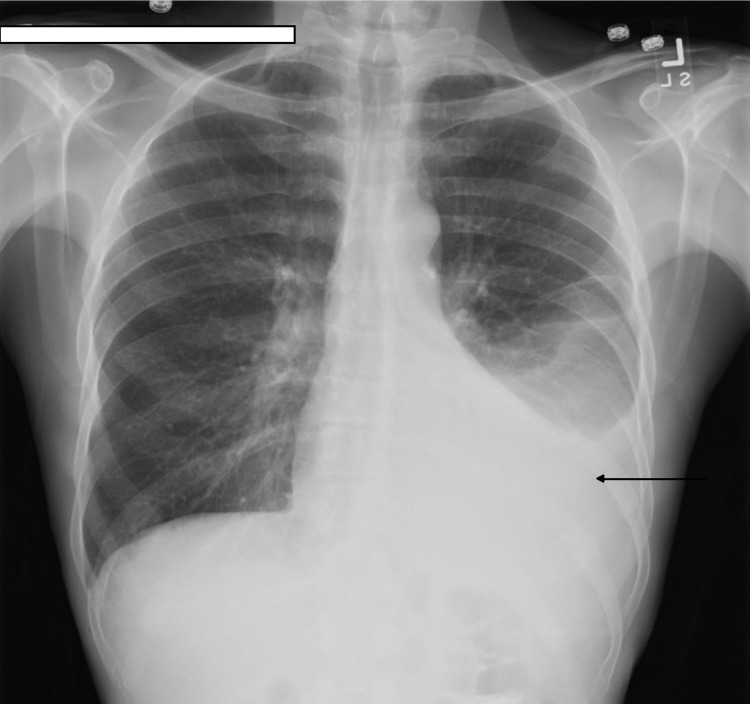
Chest X-ray shows large left side pleural effusion (arrow).

**Figure 2:**
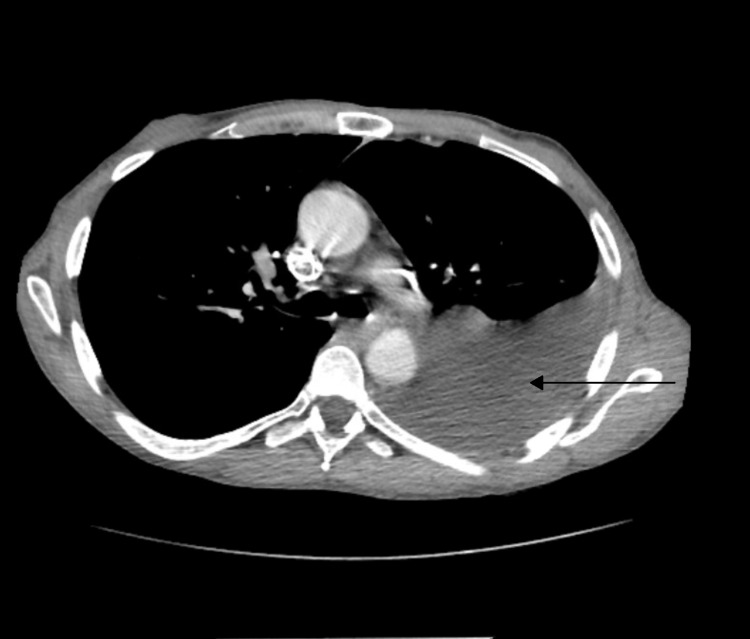
Chest CT shows left pleural effusion (arrow).

**Figure 3:**
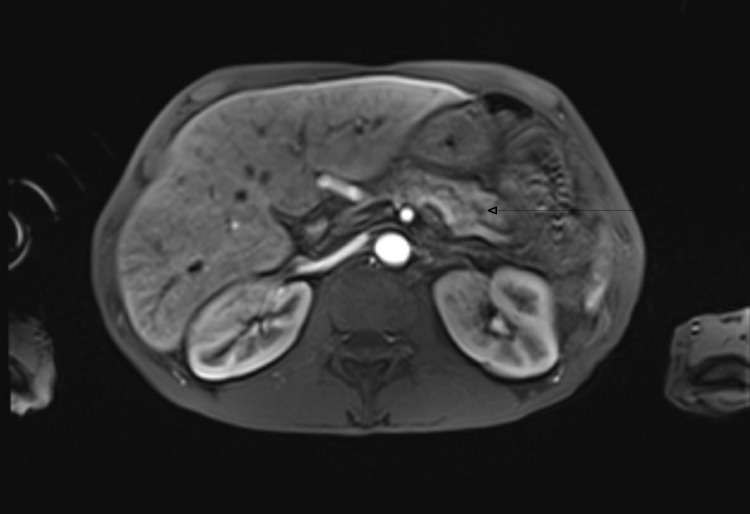
Magnetic resonance cholangiopancreatography shows findings consistent with acute pancreatitis (arrow).

**Figure 4:**
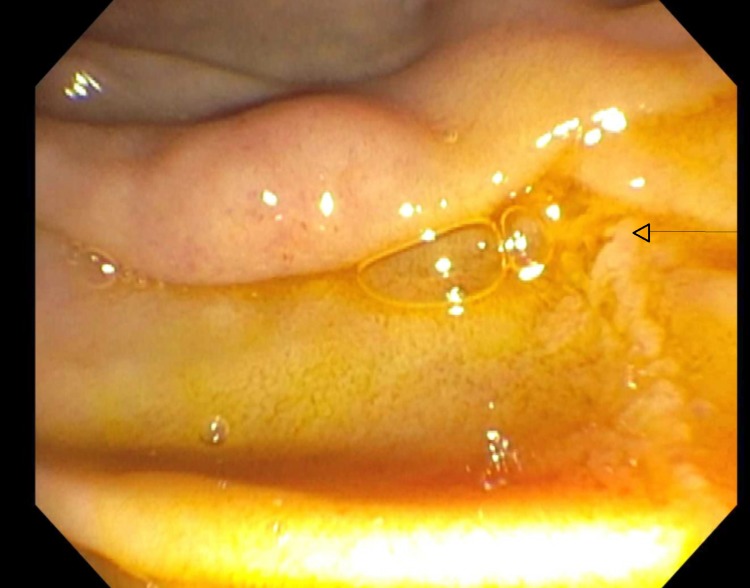
Endoscopic retrograde cholangiopancreatography showed dilated duct with evidence of complete obstruction in the body because of stone with failure of the dye to pass (arrow).

Blood work showed leukocytosis with a WBC count of 14.1X 10E9/L, serum amylase of 2636 U/L, serum lipase of 1199 U/L, serum creatinine 0.68 mg/dl, BUN 13 mg/dL, AST 22 U/L, ALT 25 U/L, alkaline phosphatase 58 U/L, and total bilirubin 0.4 mg/dL. Chest X-ray showed large left sub-pulmonic pleural effusion (Fig 1). The patient was started on IV hydration along with pain control and was not given food or water before the surgery. CT scans of the abdomen showed a dilated pancreatic duct and calcifications in the pancreas consistent with chronic pancreatitis. It also showed left pleural effusion and atelectasis of the left lower lung lobe (Fig 2). The patient underwent thoracentesis and a chest tube placement that yielded 1 L of pleural fluid. According to Light's criteria, pleural fluid was transudative with significantly elevated amylase and lipase (pleural fluid amylase of 48,466U/L, lipase >33,000U/L, pH of 7.5 triglycerides <10 mg/dl, total protein 1 g/dL, LDH of 58U/L, serum LDH 259 U/L, serum total protein 5.3 g/dL) which raised suspicion for pancreatic pleural fistula. Magnetic resonance cholangiopancreatography was showed findings consistent with acute on chronic pancreatitis along with a complex left pleural effusion (Fig 3), peritoneal fluid, and fluid in the anterior pararenal space. It was difficult to delineate the fistula between the pancreas and the pleura due to pancreatic obstruction at the level of the body of the pancreas due to a large stone. The patient then underwent ERCP with endoscopic ultrasound for further diagnosis and visualization of the pancreatic pleural fistula. A trial of stent placement in the pancreatic duct to obliterate the fistula and to decompress the pancreatic duct was conducted in order to allow the pancreatic secretions to follow the lower pressure pancreatic duct to the duodenum rather than to the pleural space. However, a large stone measuring 10 mm was noted in the pancreatic duct obstructing the view as the contrast couldn’t pass the stone; therefore, a stent was not able to be placed (Fig 4). The pancreatic duct was 7.5 mm in diameter at the body distal to stone. The head of the pancreatic duct measured 5.7 mm, and the tail of the duct measured 6.2 mm. The surgery team was consulted to evaluate the patient for possible surgical intervention, recommending starting total parenteral nutrition with surgical intervention plan within a week. The patient tolerated total parenteral nutrition well. Repeated chest X-rays prior to removal of the chest tube showed re-accumulation of the pleural fluid. The patient's symptoms of shortness of breath, abdominal pain, and nausea improved throughout his hospitalization. However, due to the obstructing stone in the pancreatic duct that was visualized during the ERCP and the rapid reaccumulating pleural effusion, the patient underwent Frey procedure with open Roux-en-Y lateral pancreaticojejunostomy, splenectomy and cholecystectomy. In the operation, there was a fistula extending from the body of the pancreas to the left diaphragm that traveled posterior to the stomach and along the esophagus via the diaphragmatic hiatus. The fistula was ligated during the surgery. The patient tolerated the surgery well, the chest tube was removed, no more pleural fluid was accumulated. The patient was discharged home with a follow-up visit to receive his vaccination post-splenectomy.

## Discussion

Pathophysiology and PPF formation

Pancreaticopleural fistula occurs in less than 1% of patients with acute pancreatitis 0.4% of patients with chronic pancreatitis and in 4.5% of patients with pseudocyst [1-4]. Pancreatic pseudocyst is defined as an abnormal collection of pancreatic secretions contained within a non-epithelial wall, that is composed primarily of granular and fibrous tissue [4]. Alcohol-related chronic pancreatitis is the leading cause of fistula formation [1-7]. However, PPF can occur as a result of gallstone pancreatitis, idiopathic pancreatitis, trauma, or congenital anomalies of the pancreatic duct. PPF can be formed due to posterior pancreatic pseudocyst rupture into the retroperitoneum, causing the pancreatic secretions to ascend to the pleural space through the esophageal or aortic hiatus, or, due to the abnormal direct transdiaphragmatic connection of the pancreatic duct to the pleural space [1, 3, 6]. PPF-related pleural effusion should be distinguished from the reactive pleural effusion that occurs with pancreatitis that is usually left-sided and self-limiting. If the pancreatic pseudocyst ruptures anteriorly, pancreaticperitoneal fistula will occur and manifests as ascites. On a very rare occasion, communication with the bronchial tree, esophagus, or the pericardium has also been reported with the latter manifesting as pericardial effusion [1-2]. 

Clinical presentation 

A typical clinical scenario for PPF is a male patient, in the mid-40s, with chronic pancreatitis secondary to alcoholism with recurrent pleural effusion that is rapidly reaccumulating and resistant to thoracentesis. Pleural effusion secondary to PPF is more common on the left (42%-67%). However, it can occur on the right side (19%-40%) or bilaterally (14%-17%) [1]. Presenting symptoms are mainly pulmonary related, with dyspnea being the most common presenting symptom in 65%-76% of the cases [1-6,8,9]. Abdominal symptoms were reported in 24% of the cases. [3]. 

Diagnosis 

Diagnosing PPF needs high clinical suspicion. As symptoms are mainly pulmonary, the diagnosis is usually delayed with the average time of diagnosis is 5 weeks [3]. Diagnosis can be established via thoracentesis with pleural fluid analysis demonstrating exudative effusion with high amylase level [1, 3, 8, 10]. There is no cut-off point for amylase level. However, it is usually significantly elevated with a mean amylase level above 10,000 U/L [1,4]. Of note, serum amylase has no role in the diagnosis [2]. Other causes of amylase-rich pleural effusion include parapneumonic effusion, pulmonary tuberculosis esophageal rupture and malignancies like leukemia, lymphoma and gynecological malignancies [1-2]. Only PPF related effusions have the pancreatic-type amylase; other causes of amylase-rich pleural effusion will have the salivary-type amylase in the pleural effusion [3]. Once pleural fluid analysis confirms the presence of high amylase level, the next step is to confirm the presence of the fistula. The most sensitive imagining modality is magnetic resonance cholangiopancreatography (MRCP) with a sensitivity of 80%, followed by endoscopic retrograde cholangiopancreatography (ERCP) with a sensitivity of 78% followed by CT scan with a sensitivity of 47% [1, 9]. ERCP is superior to other modalities as it can be diagnostic and therapeutic at the same time. However, as it is an invasive procedure with potential complications like pancreatitis, ductal perforation, and bleeding, MRCP became the diagnostic tool of choice, especially if surgical management is being considered as MRCP can provide detailed anatomy of the area [1, 7]. 

Treatment 

PPF can be treated medically, endoscopically and surgically [1-6]. Medical treatment with octreotide to decrease pancreatic secretions accompanied by total parenteral nutrition has been successful in 31%-65% of the cases. However, it needs two to three weeks of medical treatment and it is often conjugated with chest tube placement to drain the recurrent pleural effusion in that period [3, 9]. Complications of medical management may include malnutrition, central catheter infection, and sepsis [3]. Endoscopic treatment with ERCP stenting to the pancreatic duct has also been successful. ERCP stenting has two main goals: The first one is to mechanically block the abnormal connection of the pancreatic duct with the pleura; the second goal is to keep the pancreatic duct open so the pancreatic secretions can go downstream the duct of lower resistance to the duodenum thus escaping the abnormal pleural connection with the higher resistance [10]. Complications of ERCP treatment include anatomical disruption of pancreatic duct and recurrent fluid accumulation. Surgical treatment is the last resort when medical and ERCP treatment fail [1-8]. With the advanced surgical techniques, surgical treatment now has low morbidity and mortality, associated with quicker recovery and better outcome than medical or endoscopic treatment. King et al. reported 94% success rate for surgical treatment compared to 31% success rate for medical treatment. The same study also reported that medical treatment takes 50% more time than the time required for surgical recovery and that 70% of the reported surgical complications occurred in patients who were treated initially with medical management. 

PPF treatment needs multidisciplinary approach including pulmonary, gastroenterology, and general surgery teams on board. The decision to proceed with medical, endoscopic, or surgical treatment is case-dependent and takes into consideration the imagining finding of the pancreatic duct anatomy [1-7, 10]. Patients with MRCP findings of a normal pancreatic duct with no strictures can be managed medically. Those with ductal disruption in the pancreatic head or body with a stricture downstream the disruption should me managed with ERCP. Surgical treatment is indicated when there is a complete obstruction of the pancreatic duct, disruption of the pancreatic duct in the pancreatic tail, and those who are not amenable to ERCP or who failed ERCP treatment [5-7]. The main principle of surgical treatment is to form a pancreatic enteric connection to achieve adequate drainage of the pancreatic sections with or without pancreatic resection. The most common surgery reported in the treatment of PPF is distal pancreatectomy with pancreaticojejunostomy. In the case of pancreatic head mass that is compressing on both the pancreatic duct and the biliary tree, the Frey procedure can be done, which involves pancreatic head resection with longitudinal pancreaticojejunostomy [2]. 

## Conclusions

In conclusion, PPF diagnosis starts with clinical suspicion and a compatible clinical picture. Demonstrating high amylase level in the pleural fluid analysis is a key. Imaging modalities are of high value with MRCP being the most sensitive image. Treatment can be medical, endoscopic or surgical. Choosing between the options is case dependent and needs multidisciplinary teams. 
